# Novel reactions in acyl editing of phosphatidylcholine by lysophosphatidylcholine transacylase (LPCT) and acyl-CoA:glycerophosphocholine acyltransferase (GPCAT) activities in microsomal preparations of plant tissues

**DOI:** 10.1007/s00425-014-2184-1

**Published:** 2014-10-09

**Authors:** Ida Lager, Bartosz Glab, Lovisa Eriksson, Guanqun Chen, Antoni Banas, Sten Stymne

**Affiliations:** 1 Department of Plant Breeding, Swedish University of Agricultural Sciences, 230 53 Alnarp, Sweden; 2 Intercollegiate Faculty of Biotechnology of University of Gdańsk and Medical University of Gdansk, 80-822 Gdańsk, Poland; 3 Alberta Innovates Phytola Centre, Department of Agricultural, Food, and Nutritional Science, University of Alberta, Edmonton, AB T6G 2P5 Canada

**Keywords:** Lipid biochemistry, Phosphatidylcholine metabolism, Glycerophosphocholine:acyl-CoA acyltransferase, Lysophosphatidylcholine transferase

## Abstract

****Main conclusion**:**

**Plants have lysophosphatidylcholine transacylase** **(LPCT) and acyl-CoA:glycerophosphocholine acyltransferase (GPCAT) activities. The combined action of LPCT and GPCAT provides a novel route of PC re-synthesis after its deacylation.**

**Abstract:**

Phosphatidylcholine (PC) is the major lipid in eukaryotic membranes and has a central role in overall plant lipid metabolism. It is also the site of production of polyunsaturated fatty acids in plants. The recently discovered acyl-CoA:glycerophosphocholine acyltransferase (GPCAT) activity in yeast provides a novel route of re-synthesising PC via lysophosphatidylcholine (LPC) after its deacylation. This route does not require the degradation of the glycerophosphocholine (GPC) into free choline, the activation of choline to CDP-choline, nor the utilization of CDP-choline by the CDP-choline:diacylglycerol cholinephosphotransferase. We show here that GPCAT activities also are present in membrane preparations from developing oil seeds of safflower and other species as well as in membrane preparations of roots and leaves of Arabidopsis, indicating that GPCAT activity plays a ubiquitous role in plant lipid metabolism. The last step in formation of GPC, the substrate for GPCAT, is the deacylation of LPC. Microsomal membranes of developing safflower seeds utilized LPC in LPC:LPC transacylation reactions (LPCT activities) creating PC and GPC. The results demonstrate that safflower membranes have LPCT and GPCAT activities that represent novel reactions for PC acyl editing. The physiological relevance of these reactions probably has to await identification of the enzymes catalysing these reactions.

## Introduction

Phosphatidylcholine (PC) is a central molecule in overall lipid metabolism outside the plastid in plant cells. Not only is PC the substrate for producing polyunsaturated fatty acids (Bates et al. 2013) but labelling studies in pea leaves (Bates et al. 2007) and developing soybean (Bates et al. 2009) have shown that it is the main recipient for acyl groups exported from the plastids. Thus, there is a rapid acyl editing of PC. PC is the substrate for the ∆12 desaturase that desaturates oleate to linoleate. In safflower (*Carthamus tinctorius*) seeds, 80–90 % of all the fatty acids of the oil (triacylglycerols, TAG) are linoleate (Stymne and Appelqvist 1978). Thus, the acyl groups in PC in these seed cells undergo rapid turnover whereby oleate is channelled into PC for desaturation and the resulting linoleate is channelled from PC into TAG. The acyl groups can be channelled from PC to TAG via DAG by the activity of the phosphatidylcholine:diacylglycerol cholinephosphotransferase (PDCT) (Lu et al. 2009). The acyl groups can also be removed from the glycerol backbone of PC for channelling to TAG by three different types of enzymes that all produce lysophosphatidylcholine (LPC): (1) The acyl groups from PC can be removed by phospholipases (Bafor et al. 1991; Bates et al. 2013), (2) by the reverse reaction of the acyl-CoA:lysophosphatidylcholine acyltransferase (LPCAT) (Lager et al. 2013; Stymne and Stobart 1984) or (3) by the phospholipid:diacylglycerol acyltransferase (PDAT) (Banas et al. 2013). LPC is the substrate for the production of glycerophosphocholine (GPC) by phospholipases of B type but also for the re-synthesis of PC catalysed by the LPCATs (Stymne and Stobart 1984; Ståhl et al. 2008). Microsomal fractions of developing safflower seeds have very high LPCAT activity in its forward reaction in presence of acyl-CoA and added LPC (Stymne and Stobart 1984).

The recently discovered glycerophosphocholine:acyl-CoA acyltransferase (GPCAT) activity in yeast (Stålberg et al. 2008) provides a novel direct route of PC re-synthesis via lysophosphatidylcholine (LPC) following its deacylation. This route does not require the degradation of GPC into free choline, the activation of choline to CDP-choline nor the utilization of CDP-choline by the CDP-choline:diacylglycerol cholinephosphotransferase (CPT). In this work, we show that microsomal preparations from developing safflower and a number of other oil seeds have GPCAT activity. Also membranes prepared from Arabidopsis leaves and shoots possessed GPCAT activity, although at much lower levels than the oil seed membranes.

Since the microsomal preparations from developing safflower seeds showed good GPCAT activity, we investigated how added LPC was metabolized in these membranes to characterize enzymatic reactions leading to the formation of GPC, the substrate for GPCAT. Our results show that LPC added to the membranes at endogenously occurring concentrations in the microsomes is primarily utilized in transacylation reactions with another LPC molecule creating PC and GPC. We name this activity lysphosphatidylcholine transacylase (LPCT) activity according to previous suggestion (Sugimoto and Yamashita 1999). The membranes had also considerable LPC acyl hydrolase activity, and it could be speculated that enzymes of phospholipase B type could carry out both transacylase and acyl hydrolase activities (Witt et al. 1984a, b).

The results presented demonstrate that safflower membranes have LPCT and GPCAT activities that represent novel reactions in plant PC acyl editing.

## Materials and methods

### Chemicals

[1-^14^C]Radioactive fatty acids and [^14^C]choline were purchased from Perkin-Elmer, non-radioactive fatty acids and CoA were obtained from Larodan (Malmö, Sweden). Acyl-CoAs were prepared according to the method described by Sanchez et al. (1973). [^14^C]Acyl-LPC was prepared by acylation of the trifluoroacetic anhydride of the radioactive fatty acid to glycero-*sn*-3-phosphocholine (Sigma) according to Kanda and Wells (1981). The isolated [^14^C]acyl-LPC was kept in methanol solution for at least two days at room temperature to allow for isomerization to the more stable *sn*-1-LPC isomer. In the studies of the LPCT reaction it was of importance to know the ratios of the positional isomers of the LPC substrate. This was done by incubating safflower microsomes (12 µg of microsomal protein) with 10 nmol of [^14^C]18:1-LPC and 16 nmol non-radioactive 18:1-CoA in 0.1 M phosphate buffer, pH 7.2 in a total assay volume of 100 µl for 3 min at 30 °C. The radioactive PC formed (84.3 ± 3.9 % of the added [^14^C]LPC was acylated, value given from triplicate assays) was treated with PLA_2_ (from *Naja naja*, Sigma) and the distribution of radioactivity between *sn*-1 position and *sn*-2 position was determined by the ratio of radioactivity between the LPC (position *sn*-1) and free fatty acids (position *sn*-2) after separation of the products from lipase treatment by TLC. The radioactivity in PC resided to 91.5 ± 1.3 % (triplicate assays) in the *sn*-1 position, likely to reflect a positional isomer equilibrium of 9:1 between the *sn*-1 and *sn*-2 positions of LPC. It should be noted that plant LPCAT also acylate *sn*-2-LPC (Lager et al. 2013). [^14^C]Choline-labelled [^14^C]glycerol-*sn*-3-phosphocholine and [^14^C]choline-labelled LPC were obtained by growing wildtype yeast cells (SCY62) with [^14^C]choline in synthetic defined (SD) liquid minimal media at 30 °C for 24 h. The yeast cells were collected by centrifugation and disrupted by homogenization with 0.5 mm zirconia/silica beads using a Mini Beadbeater-8 (Biospec Products). The lipids were extracted into chloroform and separated by silica TLC as described below. PC was eluted from the gel and extracted into chloroform and either deacylated according to Stålberg et al. (2008) to obtain [^14^C]glycerophosphocholine or treated with PLA_2_ (*Naja naja*, Sigma) (Bafor et al. 1991) and resulting [^14^C]choline-LPC was isolated after separation on TLC. The acyl groups of the [^14^C]choline-labelled PC or LPC had no detectable radioactivity (data not shown). [^14^C]Choline-labelled PC from the yeast incubations was also treated with phospholipase C (from *Clostridium perfringens*, Sigma company*)* and the reaction mixture was extracted with chloroform/methanol/water according to Bligh and Dyer (1959) and methanol/water phase was removed and used as standard for [^14^C]phosphocholine in separation of water soluble metabolites as described below.

### Plant microsomal preparations

Microsomal membranes from developing seeds of safflower (*Carthamus tinctorius*), castor bean (*Ricinus communis*), elm (*Ulmus glabra*), Crambe (*Crambe abyssinica*) and rape seed (*Brassica napus*) were prepared according to Guan et al. (2014) and Ståhl et al. (2004). The microsomal preparations were flash frozen in liquid nitrogen and stored at −80 °C until used in the assays. Arabidopsis plants (Col 0) were grown in 100 ml sterile liquid media (0.5 × Murashige and Skoog medium supplied with 1 % sucrose) for 25 days (approximately 70 seeds per flask). Seedlings were split in root and leaves and microsomal preparations were prepared according to Guan et al. (2014), flash frozen with liquid nitrogen and stored at −80 °C until used in the assays.

### Enzyme assays

GPCAT activity assays were performed with washed microsomal membranes from developing seeds equivalent to 40 µg of microsomal protein, 25 nmol of [^14^C]GPC (specific activity 4,900 dpm/nmol), 10 nmol of acyl-CoA, 1 mg BSA and 10 mM EDTA in 0.1 M phosphate buffer, pH 7.2 in a final volume of 100 µl at 30 °C at times indicated in the figures. GPCAT activity in safflower membranes was also assayed with radioactive [^14^C]16:0-CoA and non-radioactive GPC at the same concentration of microsomes and substrates, BSA and EDTA as described above. In interpreting the results of these assays, it was essential that no radioactive acyl group entered PC via acylation of endogenous LPC or via acyl exchange. Therefore, the assays also contained 0.5 mM dithionitrobenzoic acid (DTNB) to inhibit the acyl exchange (Lager et al. 2013) and the microsomes were pre-incubated with 2 nmol of 18:1-CoA before addition of GPC and [^14^C]16:0-CoA to acylate endogenous LPC with unlabelled 18:1. Incubations with [^14^C]16:0-CoA but in the absence of added GPC showed no significant radioactivity in PC (Fig. 3).

LPCT activity was measured with plant microsomal preparation by adding 4 nmol of either [^14^C]acyl-LPC or 4 nmol acyl-[^14^C]choline-LPC dissolved in 20 µl water to membranes corresponding to 360 µg of microsomal protein in 0.1 M phosphate buffer, pH 7.2 under vigorous vortexing for 30 s whereafter the incubation proceeded with shaking (250 rpm/min) at 30  C for times indicated in the figures.

### Lipid extraction, separation and analysis

Lipids from microsomal preparation of safflower seed were extracted into chloroform (Bligh and Dyer 1959) and aliquots of the chloroform phase were separated on silica thin layer chromatography (TLC) plates (Merck silica 60) in both polar solvent system (chloroform:methanol:acetic acid:water, 90:15:10:3, v/v/v/v.) and neutral solvent system (heptane:diethylether:acetic acid, 70:30:1, v/v/v) to separate LPC and PC (polar system) and diacylglycerol (DAG) (neutral system). The plates were sprayed with 0.05 % primulin according to White et al. (1998) and PC, LPC and DAG were identified under UV light by *R*_*f*_ values of authentic standards. The gel areas with the lipids were removed and methylated in situ and the fatty acid content and composition was determined by GLC using 17:0 methyl ester as an internal standard, as described below.

The microsomal assays were terminated by addition of 100 µl of 0.15 M acetic acid and 500 µl chloroform:methanol (1:1, v/v.) and vortexed. After centrifugation, the lower (chloroform) layer was removed and an aliquot was taken to liquid scintillation counting of the radioactivity. The rest of the lower phase was applied on silica TLC plate (silica 60, Merck) and the plate was developed in polar solvent; chloroform:methanol:acetic acid:water (90:15:10:3, v/v/v/v.) to the top in case of GPCAT assays (10 cm high plates) or to half its height in case of LPCT assays (20 cm high plates). In the latter case, the TLC plates were dried under a stream of nitrogen and re-developed in heptane:diethylether:acetic acid (70:30:1, v/v/v) to the top. Radioactive spots were visualized and identified by *R*_*f*_ values of authentic radioactive standards and the relative amount of radioactivity in each spot was determined by Instant Imager (Packard Canberra) electronic autoradiograph. Absolute amounts of radioactivity in each spot were calculated from the total amount of radioactivity in the chloroform phase as determined by liquid scintillation.

Analysis of radioactivity at the different *sn*-positions of [^14^C]PC isolated after assays with [^14^C]acyl-LPC was performed by hydrolysis with phospholipase A_2_ (from *Naja naja*, Sigma) according to Bafor et al. (1991). The products from hydrolysis were separated in the polar TLC system as described above and the relative radioactivity in [^14^C]LPC (position *sn*-1) and (^14^C)-free fatty acids (position *sn*-2) was determined by Instant Imager. When a mixture of [^14^C]16:0-LPC and [^14^C]18:2-LPC was used in the assays, the free fatty acids and LPC were scraped off the plates, methylated as described below and methyl esters were separated on silver nitrate impregnated TLC plates in heptane:diethylether:acetic acid (85:15:1). The relative distribution of [^14^C]16:0 and [^14^C]18:2 in LPC and free fatty acids was determined by Instant Imager.

When water soluble metabolites were analysed in assays with acyl-[^14^C]choline-LPC, 5 µl of trichloroacetic acid was added to the methanol/water phase from the extraction of the assays and centrifuged at 15,000 rpm for 10 min to remove denatured proteins. The supernatant was evaporated to dryness over nitrogen, the residue was dissolved in 5 µl water and 1 µl was spotted on cellulose TLC plates (Merck), which was then developed in ethanol: 2.7 M ammonium acetate, pH 5 (7:3, v/v).

### Fatty acid analysis

Fatty acids were analysed and quantified by GC analysis after conversion to corresponding methyl esters by heating in 2 ml of 2 % H_2_SO_4_ in water free methanol in capped tubes at 90 °C for 30 min. The methyl esters were then extracted into hexane by adding hexane (2 ml) and water (2 ml). GC analysis of fatty acid methyl esters was performed on a CP-wax 58 (FFAP-CB) column using a Shimadzu gas chromatograph. The identification of fatty acid methyl esters was performed by comparing the retention times with authentic standards. Quantification of fatty acid methyl esters was done by addition of heptadecanoic acid methyl esters as internal standard.

### Statistical analysis

Enzyme assays were done in triplicate and values are shown as ± SD, unless stated otherwise in the figure legend.

## Results

### GPCAT activity in plants

**Fig. 1:**
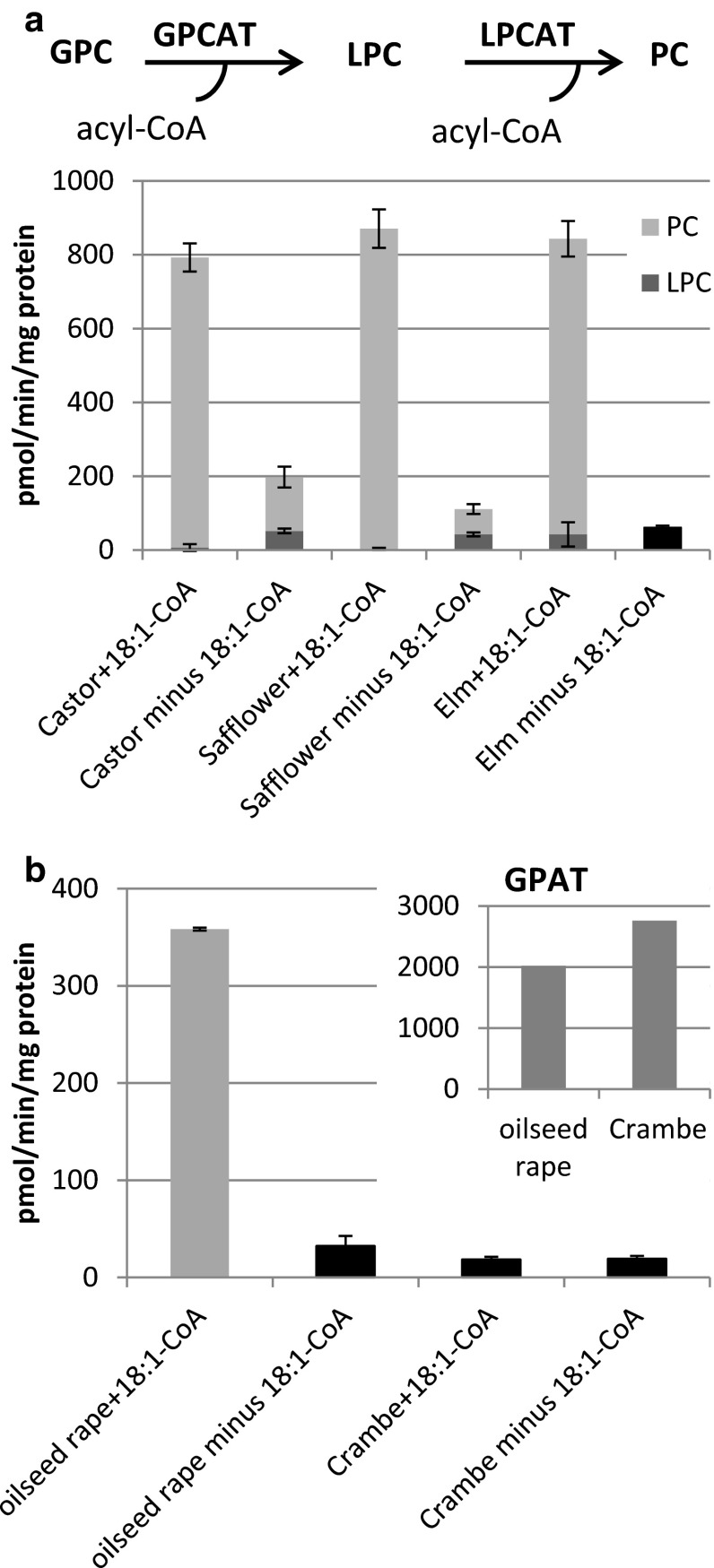
GPCAT activities in microsomal preparations from various developing oil seeds. The *dark grey* part of the bar represents the amount of radioactive LPC formed and the *light grey* part the amount of radioactive PC formed. Due to too low activity in membranes from Crambe and elm seed and rape in the absence of added acyl- CoA it was not possible to get accurate readings of the proportions of radioactivity between PC and LPC and the *black bars* represent the total amount of radioactivity incorporated into the chloroform fraction (the [14C]glycerophosphocholine substrate resides exclusively in methanol/water phase after extraction). **a** GPCAT activity with and without addition of 18:1-CoA. The combined enzyme reactions of LPCAT and GPCAT in the microsomal preparations are depicted at the top of the figure. **b** GPCAT and GPAT (*inserted diagram*) activities in microsomal preparations from developing seeds of oil seed rape (*Brassica napus*) and *Crambe abyssinica* in the presence and absence of 18:1-CoA. Microsomal membranes were re-suspended in buffer containing 0.1 % BSA and re-pelleted before used in assays. Microsomes corresponding to 40 μg were incubated with 25 nmol of [^14^C]glycerophosphocholine (specific activity 4,750 dpm/nmol) or 10 nmol of [14C]glycerol 3-phosphate (specific activity 6,100 dpm/nmol) for 20 min at 30 °C. All GPCAT assays were done in triplicate and values are shown ± SD

Microsomal membranes prepared from developing seeds of castor bean, safflower and elm were incubated with [^14^C]choline-labelled [^14^C]GPC and 18:1-CoA (Fig. 1a). The LPCT activity assays, as described below, showed that the safflower microsomes contained no or only very low amount of endogenous acyl-CoA. However, to make sure that all acyl-CoAs were removed from the membranes in the GPCAT assays, they were re-suspended in large volumes of phosphate buffer containing 0.1 % BSA and re-pelleted by ultracentrifugation before used in GPCAT activity assays. All these membrane preparations had the capacity to acylate GPC into radioactive PC in the presence of added acyl-CoA (Fig. 1a). The product of acylation of GPC is LPC, but since oil seed microsomal membranes contain high LPCAT activities, the formed LPC will immediately be acylated into PC. Thus, radioactive PC was the only chloroform soluble product with significant radioactivity in assays with safflower, castor bean and rape seed membranes (Fig. 1b) in the presence of acyl-CoA. Surprisingly, significant amount of GPCAT activity could also be seen in absence of added acyl-CoA, varying from 5 % of the activity with addition of acyl-CoA in membranes from elm seeds to 12 and 25 % in membranes from safflower and castor bean, respectively (Fig. 1a). Even more intriguingly, even in the absence of acyl-CoA, the main radioactive product was PC but a substantial portion of the radioactivity was also found in LPC in assays with both castor bean and safflower membranes (Fig. 1a).

We also assayed the GPCAT activity in presence of added 18:1-CoA in membranes prepared from developing seeds from oil seed rape (*Brassica napus*) and *Crambe abyssinica* both belonging to the *Brassicaceae* family. Whereas the rape membranes had about half the GPCAT activity of the membranes from castor, safflower and elm, the activity in Crambe membranes was just on the border of being significant (Fig. 1b). Both membranes had, however, about similar glycerol 3-phosphate acyltransferase (GPAT) activities (Fig. 1b), indicating that developing Crambe seeds had very low, if any, GPCAT activity.

**Fig. 2:**
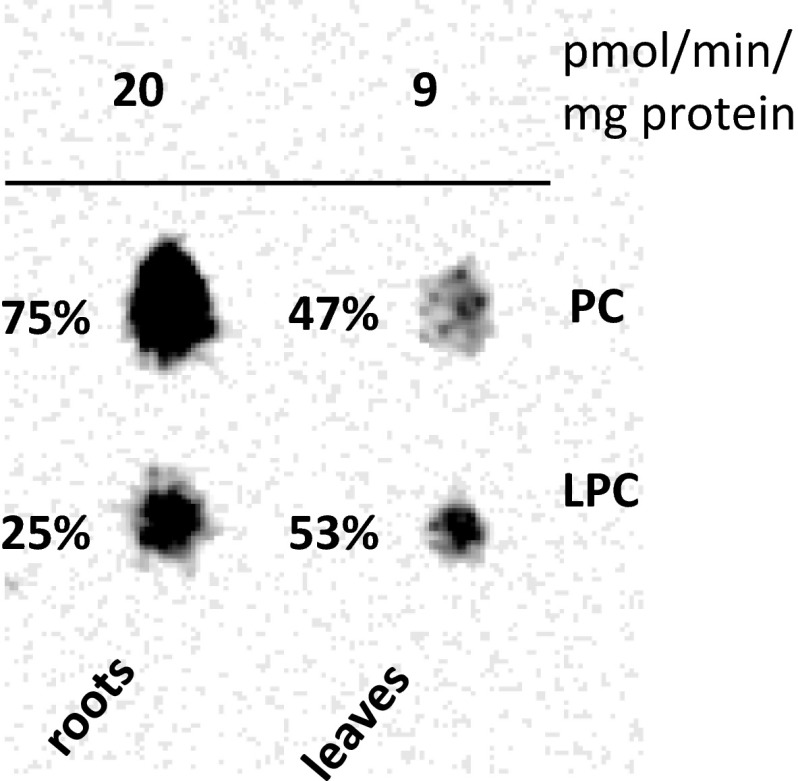
Electronic autoradiogram of lipids separated on TLC from incubations of microsomal preparations of Arabidopsis roots and shoots with [^14^C]GPC and 18:1-CoA showing the formation radioactive LPC and PC. The *number* above the *spots* shows the approximate activities in pnmol/min per mg microsomal protein. The samples applied on the TLC were from five pooled assays of 100 µg microsomal protein incubated with 25 nmol of [^14^C]glycerophosphocholine (specific activity 12,250 dpm/nmol) and 10 nmol 18:1-CoA for 30 min at 30 °C

We could also demonstrate GPCAT activities in microsomal membrane preparations from Arabidopsis roots and leaves (Fig. 2) although at much lower activity than the oil seed membranes showing this activity. In these assays substantial amount of radioactivity was also found in LPC, indicating much lower LPCAT activities in these membranes than in the oil seeds. We did not attempt to measure GPCAT activities in developing Arabidopsis seeds and thus, we do not know if the lower activities in roots and leaves of Arabidopsis are also reflected in their seeds.

**Fig. 3:**
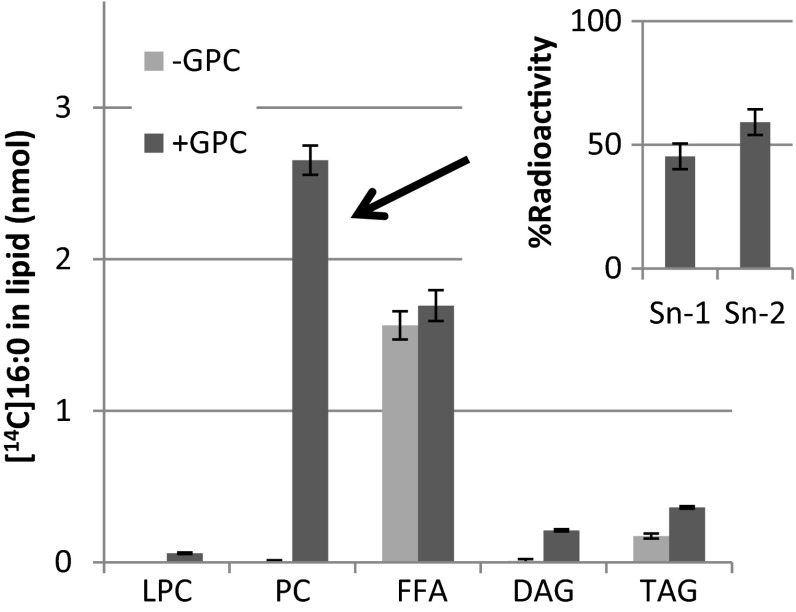
Incorporation of [^14^C]16:0 from [^14^C]16:0-CoA in lipids in the absence and presence of GPC in microsomal preparations from developing safflower seeds. Microsomal preparations corresponding to 40 µg of microsomal protein were first pre-incubated with 2 nmol non-radioactive 18:1-CoA in 0.5 mM dithionitrobenzoic acid (DTNB) for 5 min in order to acylate endogenous LPC and to inhibit acyl exchange after which only [^14^C]16:0-CoA (10 nmol) was added (*light grey bars*), or added together with 25 nmol non-radioactive glycerophosphocholine (*dark grey bars*). The inserted diagram shows the distribution of the [^14^C]16:0 radioactivity between the *sn*-positions in PC formed in the presence of GPC. Chloroform extracts from two assays with GPC were pooled to purify PC for positional analysis. All assays were done in triplicate and the *error bars* show ± standard deviation. *GPC* glycerophosphocholine, *PC* phosphatidylcholine, *FFA* free fatty acids, *DAG* diacylglycerols, *TAG* triacylglycerols

Since added radioactive GPC was converted into LPC and PC to significant extent also in the absence of added acyl-CoA, it can be speculated that the added acyl-CoA was not directly acylated to GPC but in some way stimulated the acylation of endogenous acyl groups to GPC. We therefore performed an experiment to investigate this. In this experiment, it was essential that added radioactive acyl-CoA was prevented to enter PC by other reactions than acylation of added GPC. By pre-incubating the microsomes with unlabelled 18:1-CoA, we acylated the endogenous LPC pool to prevent it to be acylated by the added radioactive acyl-CoA. We also added 0.5 mM DTNB in the assay. DTNB reacts with free CoA and since free CoA is essential for the reverse reaction of LPCAT (Lager et al. 2013), it efficiently prevented any acyl groups from added acyl-CoA to enter PC via acyl exchange. We then added [^14^C]16:0-CoA in the absence or presence of non-radioactive GPC. In the absence of GPC, no significant radioactivity was entering PC and all radioactivity found in the chloroform phase was found as free fatty acids and triacylglycerols with free fatty acids representing about 90 % of the radioactivity (Fig. 3). In the presence of non-radioactive GPC, radioactive PC was efficiently synthesised, accounting for about 55 % of the radioactivity in the chloroform phase. About equal amount of radioactive free fatty acids was formed as in the assays with no addition of GPC. Significant amount of radioactive DAG was seen in the presence of GPC and the amount of TAG was about twice that formed in absence of GPC. We also performed positional analysis of the distribution of the radioactivity in the formed PC and showed that the [^14^C]16:0 was distributed nearly equally between the two *sn*-positions (Fig. 3, inserted diagram). Since no radioactive acyl groups were found in other chloroform soluble lipids than free fatty acids and triacylglycerols in the absence of added GPC and the radioactive PC synthesised with added non-radioactive GPC had about similar radioactivity in both sn-positions, this PC must have been derived from direct acylation of added GPC with the added [^14^C]16:0-CoA, first by GPCAT activity and the second acylation presumable by LPCAT activity. The small amount of radioactivity in TAG in the absence of GPC probably represents acylation of endogenous DAG with added acyl-CoA catalysed by DGAT enzymes. The formation of radioactive DAG in presence of GPC is likely to be the result of PDCT activity where radioactive acyl groups in PC are transferred to DAG via phosphocholine exchange between the two molecules. The increase of TAG in the presence of GPC is likely to be a result of PDAT activity, acylating mainly non-radioactive DAG with radioactive acyl groups from PC.

**Fig. 4:**
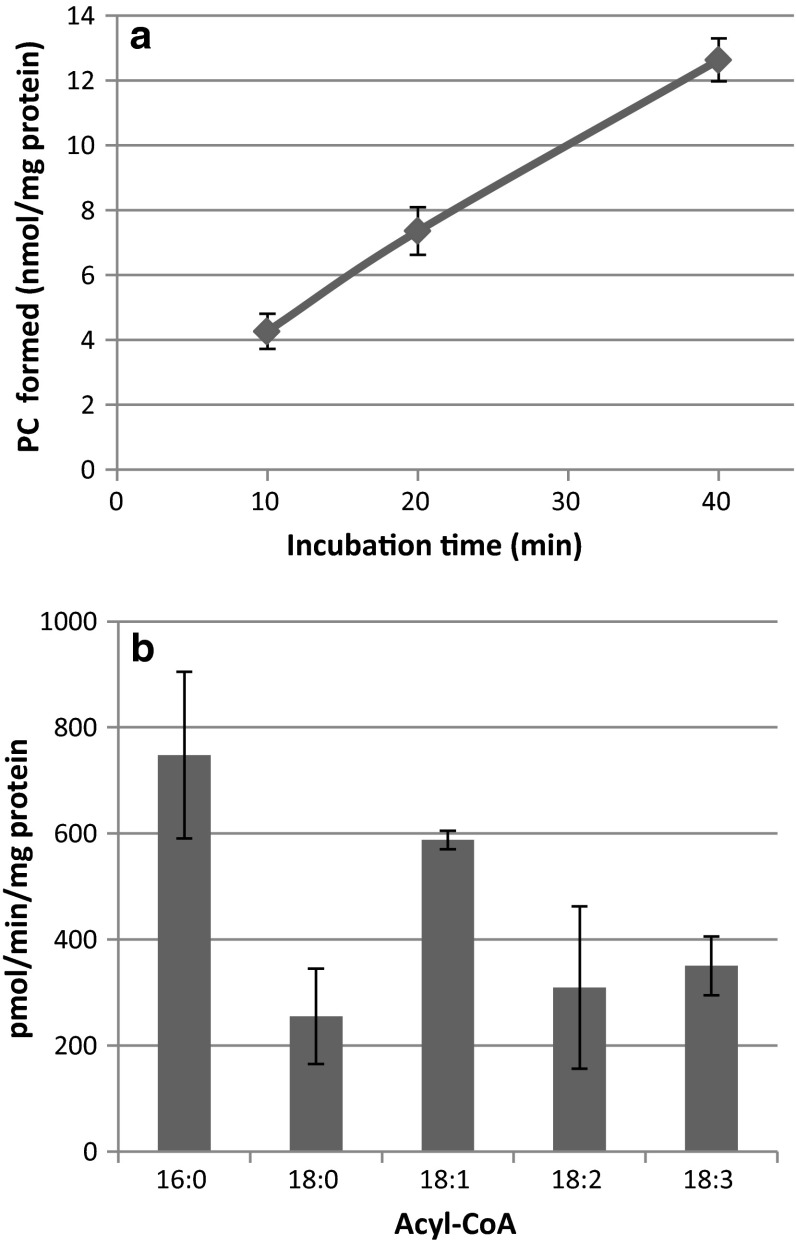
GPCAT activities in microsomal preparations from developing safflower seeds. **a** Time course of [^14^C]PC formation upon incubation of microsomal preparations of developing safflower seeds with [^14^C]glycerophosphocholine and 18:1-CoA. **b** Acyl-CoA specificity of GPCAT in microsomal preparations of safflower. Microsomal preparations corresponding to 40 µg of microsomal protein were incubated with acyl-CoAs (10 nmol) and [^14^C]glycerophosphocholine (25 nmols) for various times (**a**) or for 20 min (**b**). All assays were done in triplicate and values are shown ± SD

Time-course assays of GPCAT activities with microsomal preparations from safflower seeds supplied with 18:1-CoA showed that PC production occurs in a near-linear fashion over 40 min (Fig. 4a). We then investigated the acyl specificity of the GPCAT in these membranes using [^14^C]GPC in combinations with palmitoyl(16:0)-CoA, stearoyl(18:0)-CoA, 18:1-CoA, linoleoyl(18:2)-CoA and linolenoyl(18:3)-CoA. All these acyl groups were utilized by GPCAT, with a preference for 16:0-CoA and 18:1-CoA (Fig. 4b). It should be noted that PC was the only chloroform soluble detected radioactive metabolite in all the assays, showing that GPCAT and not LPCAT was the limiting step in the formation of PC. Thus, the amount of PC formed with the different acyl-CoA substrates reflects the GPCAT activities.

### LPC metabolism in safflower microsomes

Since the above results demonstrated that membranes prepared from various plant tissues contain GPCAT activity, the question arose as to how the GPC is formed in these tissues. Since the last step in formation of GPC is deacylation of LPC, we studied how LPC was metabolized in microsomal membranes of safflower seeds. We first determined the content and acyl chain composition of PC, diacylglycerol (DAG) and LPC in the safflower membranes to calculate the endogenous pool of these lipids that was present in the assays (Table 1).

**Table 1:** Content and acyl composition of phosphatidylcholine (PC), diacylglycerol (DAG) and lysophosphatidylcholine (LPC) in microsomal membranes prepared from developing safflower seeds

mol/mg protein	PC	DAG	LPC
	234 ± 20	55.6 ± 3.5	14.0 ± 0.60
Fatty acid composition (mol %)			
16:0	15.2 ± 0.1	16.9 ± 0.4	20.8 ± 0.2
18:0	2.3 ± 0.1	4.2 ± 0.2	3.3 ± 0.6
18:1	6.4 ± 0.3	8.0 ± 0.5	3.8 ± 0.1
18:2	75.9 ± 0.2	70.7 ± 0.2	71.9 ± 0.7

All analyses were done in duplicate and the error bars show ± SD

### LPCT activity in safflower microsomes

**Fig. 5:**
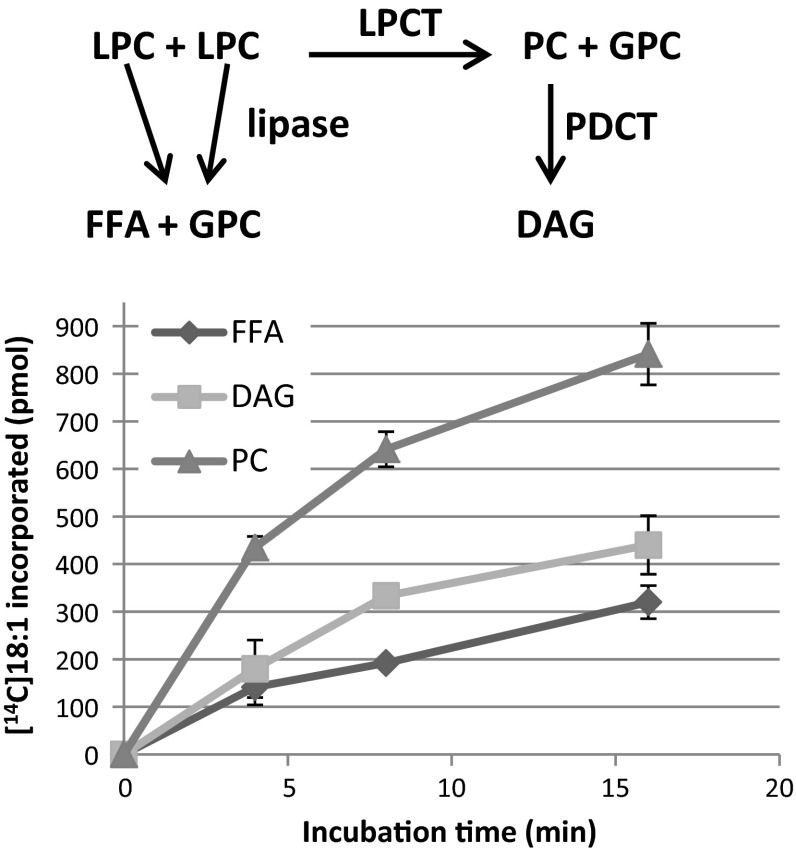
Time-course metabolism of *sn*-1-[^14^C]18:1-LPC by developing safflower microsomes. The different enzyme reactions likely to be responsible for the production of the metabolites are depicted at the top of the figure. Microsomal membranes (360 µg of protein) were incubated with 4 nmol of *sn*-1-[^14^C]18:1-LPC at 30 °C for the times indicated in the figure. All assays were in triplicate and the *error bars* show ± SD. *FFA* free fatty acids, *PC* phosphatidylcholine, *DAG* diacylglycerols, *LPCT* lysophosphatidylcholine transacylase, *PDCT* phosphocholine:diacylglycerol cholinephosphotransferase

Microsomal membranes prepared from developing safflower seeds were then incubated with *sn*-1-[^14^C]18:1-LPC. When the amount of added LPC was much higher than the amount of endogenous LPC levels in the membranes, mainly free fatty acids were formed (results not shown). However, when the amount of added LPC was similar to the endogenous levels (14 nmol/mg microsomal protein, Table 1) the main radioactive metabolite was PC, which increased in amount in a non-linear fashion during 16 min incubation (Fig. 5). The second most labelled lipid was DAG, having about half as much radioactivity as PC after 16 min of incubation. Radiolabelled free fatty acids were also formed, amounting to about 25 % of all the metabolites after 16 min. No significant radioactivity was found in any other metabolites other than those three lipids. Assays with heat treated membranes (80 °C for 5 min) showed no metabolism of added LPC (results not shown). The hypothetic enzyme reactions giving raise to these metabolites are depicted in Fig. 5 and are: LPC acyl lipase yielding free fatty acids, LPC:LPC transacylase (LPCT) giving rise to PC and GPC and phosphatidylcholine:diacylglycerol cholinephosphotransferase (PDCT) (Lu et al. 2009) activity giving rise to DAG derived from PC. The latter enzyme has previously been shown to have high activity in membranes from safflower seeds (Guan et al. 2014; Stobart and Stymne 1985). The lipase reaction will create one molecule of GPC from one molecule of LPC and LPCT reaction will produce one GPC from two LPC molecules. Since the radioactive DAG formed in the assays are with all likelihood formed from PC (formed via LPCT reaction) by the action of PDCT, it can be calculated that LPCT would account for 60 % of the GPC formed in these assays and thus would be the major reaction forming GPC.

**Fig. 6:**
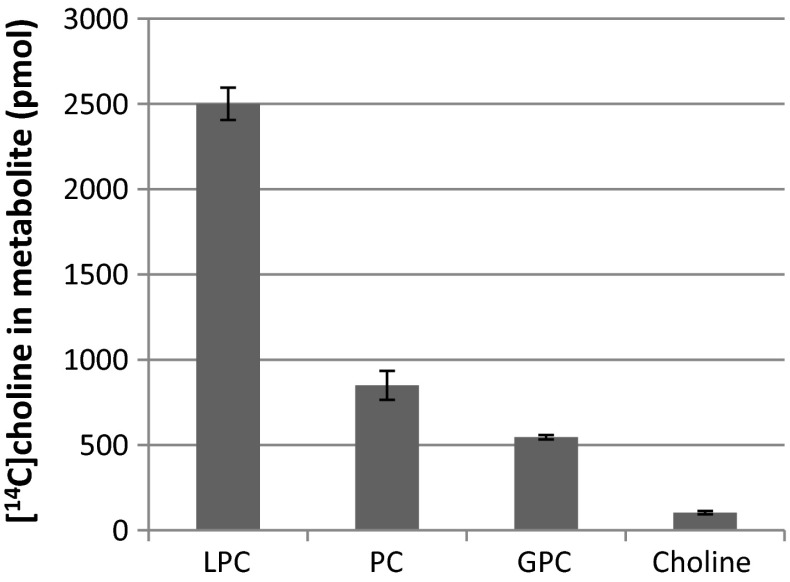
Metabolites from incubation of [^14^C]choline-labelled lysophosphatidylcholine (4 nmol) with microsomal membranes from developing safflower seeds (360 µg of protein) for 16 min. All assays were in triplicate and the *error bars* show ± SD. *LPC* lysophosphatidylcholine, *PC* phosphatidylcholine, *GPC* glycerophosphocholine

If the PC formed from added *sn*-1-LPC is synthesised by LPCT activity, this reaction should release GPC. To investigate this possibility, we incubated the safflower microsomes with [^14^C]choline-labelled LPC prepared from PC isolated from yeast that had been grown in the presence of [^14^C]choline. The only chloroform soluble radioactive compounds found in the assays with safflower membranes were the substrates LPC and PC, the latter lipid corresponding to about 20 % of the added [^14^C]LPC (Fig. 6). We also separated the compounds from the water soluble fraction by cellulose TLC. The main water soluble radioactive metabolite was GPC, in amounts corresponding to about 65 % of the [^14^C]PC formed (Fig. 6). Significant amounts of radioactive choline were also formed during incubation, amounting to 12 % of the PC formed (Fig. 6). In a separate experiment, it was found that the safflower microsomes hydrolysed the added [^14^C]choline-labelled GPC to yield free [^14^C]choline (data not shown). Thus, it is likely that the [^14^C]choline was released from the [^14^C]GPC formed in the LPC transacylation reaction via a glycerophosphodiesterase such as those identified in yeast and plants (Cheng et al. 2011; Fernandez-Murray and McMaster 2005; Fisher et al. 2005). However, it cannot be excluded that some PLD activity also was involved in the formation of the [^14^C]choline. PLD activity would have produced radioactive PA in the transacylation assays where LPC with radioactive acyl groups was added, but this was not seen (Fig. 5). However, safflower microsomal membranes contain phosphatidic acid phosphatase activities (Guan et al. 2014) and thus radioactive PA formed could have been converted to radioactive DAG, a product that was formed in the transacylation assays (Fig. 5). No radioactive phosphorylcholine was formed during the assay. In a separate experiment it was shown that the membranes did not catabolise added [^14^C]choline-labelled phosphorylcholine (results not shown). This suggests the absence of PLC activities in these membranes.

It should be noted that DAG formed from [^14^C]choline-labelled PC by PC-DAG interconversion by PDCT will not produce any new detectable radioactive lipid in our assays. The PDCT reaction would involve a transfer of the radioactive phosphocholine group from PC to DAG, which creates a new labelled PC molecule and an unlabelled new DAG molecule (Lu et al. 2009). The experiments with [^14^C]choline-labelled LPC clearly demonstrated that the added LPC is both the recipient and the donor of acyl groups to form PC. The experiments show that GPC is liberated in this process although to lesser extent than if only the added [^14^C]LPC had participated in the transacylation, even if the amount of free [^14^C]choline is included. It is likely that some free fatty acids were formed by LPC acyl lipase activity, although this could not be monitored since the acyl group of LPC was unlabelled. For each molecule of fatty acid released from the [^14^C]choline-LPC, one molecule of GPC will be formed. If we assume that the same amount of free fatty acids was formed in these assay as with [^14^C]16:0-LPC (16 % of added radioactivity, Fig. 6) the nmol GPC and choline correspond to about 60 % of the PC and free fatty acids formed. This discrepancy might be due to that the [^14^C]choline-labelled LPC prepared from yeast was considerably better acyl acceptor than donor compared to the endogenous LPC. The yeast [^14^C]choline-LPC substrate consisted mainly of 16:0 and 16:1 acyl groups (14 and 70 mol %, respectively, results not shown). The properties of 16:1-LPC in the transacylation were, however, not investigated. Other reactions acylating GPC e.g. those seen in the GPCAT activity assays without addition of acyl-CoA (Fig. 1) are also likely to contribute to the non-stoichiometric proportions of GPC and PC. Possible such reaction could be the reverse reaction of LPCT activity, re-acylating GPC with acyl groups from endogenous PC or transfer of acyl groups from endogenous PC to added LPC.

**Fig. 7:**
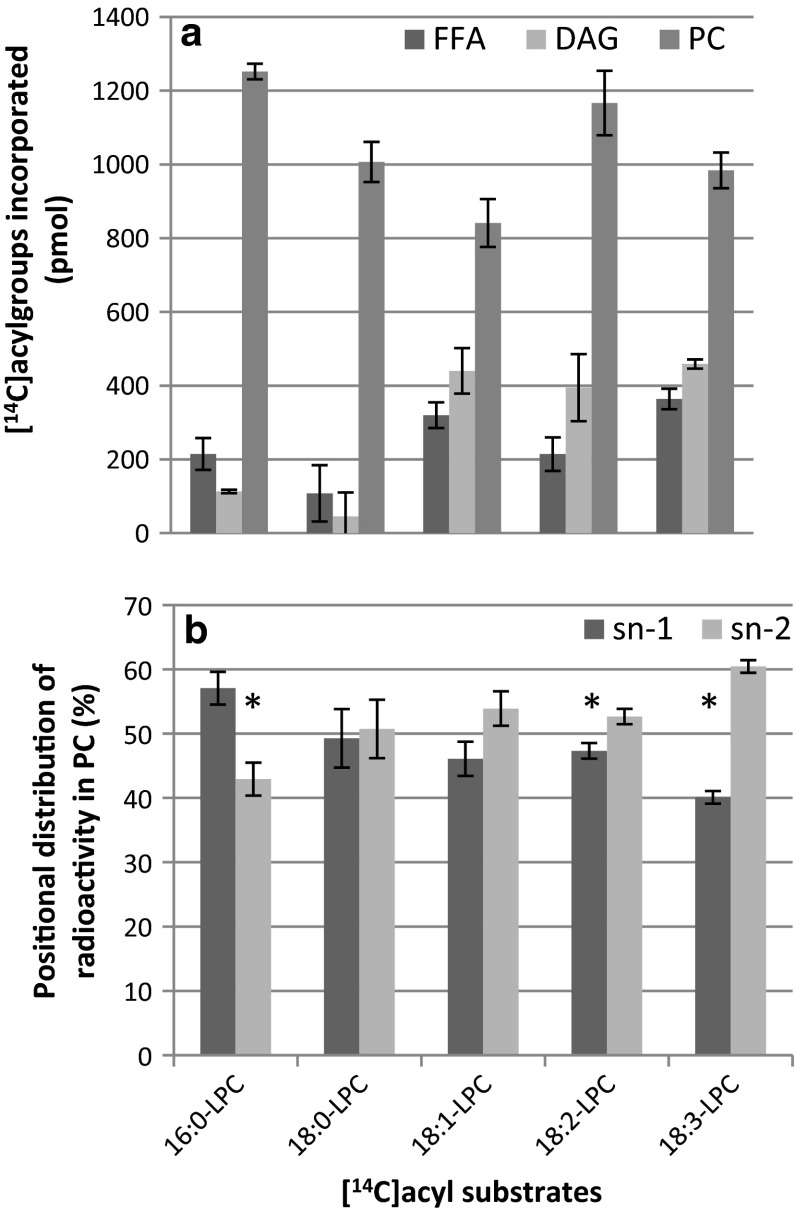
Metabolism of lysophosphatidylcholine (LPC) with various [^14^C]acyl groups by developing safflower microsomes. **a** Distribution of radioactivity in different lipid classes. **b** Positional distribution of radioactivity in the formed [^14^C]phosphatidylcholine from incubations with the various *sn*-1-[^14^C]acyl-LPC substrates. Microsomal membranes (360 µg of protein) were incubated with 4 nmol of *sn*-1-[^14^C]acyl-LPC for 16 min at 30 °C. All assays were in triplicate and the error bars show ± SD. Significant differences (*T* test, *P* < 0.05) between *sn*-*1* and *sn*-*2* are denoted with an asterisk. *PC* phosphatidylcholine, *DAG* diacylglycerol, *FFA* free fatty acids

The metabolism of radioactive *sn*-1-18:1-LPC with LPC containing palmitoyl (16:0), stearoyl (18:0), linoleoyl (18:2) and linolenoyl (18:3) acyl groups was then compared (Fig. 7a). All acyl groups were incorporated into PC to a similar extent. However, the amounts found in DAG were 75–90 % lower with the saturated acyl groups than with the unsaturated, indicating a discrimination of PDCT towards PC species with saturated fatty acids. The formation of free fatty acids was also much lower with the 18:0 substrate than with the other substrates.

It could be speculated that the radioactive PC formed was derived from acylation of added LPC with endogenous acyl-CoA catalysed by the LPCAT. We therefore determined the positional distribution of radioactivity in the *sn*-1 and *sn*-2 position of the formed PC when providing different acyl groups (Fig. 7b). About equal amounts of ^14^C- label were found in the two *sn*-positions with 18:0 and 18:1 substrates whereas 16:0 had substantially higher amounts of radioactivity in positions *sn*-1 and 18:2-LPC and 18:3-LPC had higher amounts in the *sn*-2 position. The added LPC was to over 90 % *sn*-1-LPC isomer (see “Materials and methods” section) and acylation of this LPC with endogenous (and thus non-radioactive) acyl-CoA via LPCAT would result in PC where over 90 % of the radioactivity was in the *sn*-1 position. It can thus be concluded that endogenous acyl-CoA acylated to the added LPC by LPCAT can only contribute to a minor extent in the synthesis of the radioactive PC in these membranes.

**Fig. 8:**
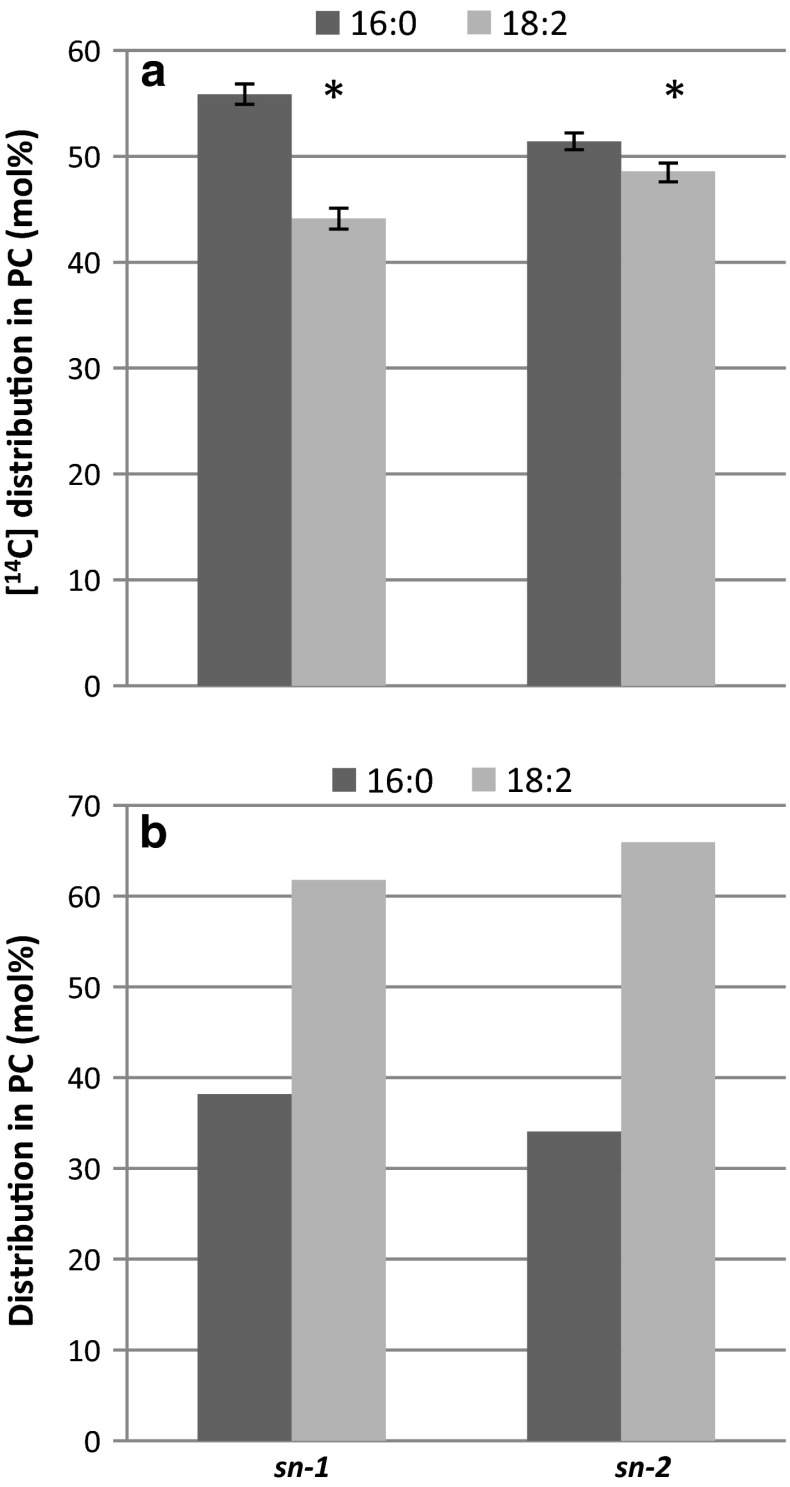
Distribution of [^14^C]16:0 and [^14^C]18:2 in the different *sn*-positions of PC formed from a mixture of equimolar amounts of [^14^C]16:0-LPC and [^14^C]18:1-LPC (2 nmol each) upon incubation with safflower microsomes (360 µg microsomal protein). **a** Molar  % distribution-based specific activity of added substrates. Two assays carried out in triplicate were pooled in each analysis and values are given as ± SD. Significant differences (*T* test, *P* < 0.05) between [^14^C]16:0 and [^14^C]18:2 are denoted with an *asterisk*. **b** Molar positional distribution of formed PC based on calculated specific radioactivity, including the endogenous 16:0-LPC and 18:2-LPC in the membranes

The most likely reaction behind the formation of PC is a transacylation between two LPC molecules that results in the formation of PC and GPC. This should result in equal amounts of the supplied radioactive LPC substrate in both *sn*-positions of PC, which was not seen with 16:0-LPC and 18:3-LPC. However, the membrane preparations also contain endogenous LPC in about the same amounts as the added LPC, with about 21 mol % of 16:0 and 72 mol % 18:2 as dominating fatty acids (Table 1). Thus, the added radioactive LPC substrate (4 nmol) was diluted 2.2 times with endogenous unlabelled substrate. If LPC:LPC transacylation activity is the mechanism for the synthesis of PC, the results depicted in Fig. 7b implies that [^14^C]16:0-LPC is a preferred acyl acceptor for acyl groups from endogenous LPC whereas [^14^C]18:3-LPC is a preferred acyl donor to the LPC pool. To further investigate the positional analysis we carried out assays with an equimolar mixture of [^14^C]16:0-LPC and [^14^C]18:2-LPC and measured the incorporation of radioactive acyl groups into the different *sn*-positions in the formed PC. When the calculation was based on mol % of added ^14^C specific radioactivity of the substrates, the results show 1.3 times higher mol % of 16:0 than of 18:2 in position *sn*-1, whereas the two fatty acids were in about equal amount in the *sn*-2 position (Fig. 8a). When taking into account the dilution of the ^14^C-substrate with endogenous 16:0-LPC and 18:2-LPC, about 62 and 66 mol % of the fatty acids in positions *sn*-1 and *sn*-2 were derived from 18:2-LPC, respectively (Fig. 8b). Thus, the higher amount of radioactive palmitic acid seen in position *sn*-1 of PC with only [^14^C]16:0-LPC as substrate (Fig. 7b) was confirmed to be due to 16:0-LPC being a better acyl acceptor in the transacylase reaction than the 18:2-LPC, the dominating endogenous molecular species of LPC.

## Discussion

We show in this work that membranes prepared from developing oil seeds from diverse species as well as roots and leaves from Arabidopsis contain GPCAT activity, as was previously described for yeast membranes. Since PC is delivering polyunsaturated fatty acid to TAG synthesis in some seeds, and thus undergoes rapid acyl chain turnover, GPCAT might be involved in re-synthesising PC after that molecule has delivered its acyl groups to TAG synthesis. This would represent a novel mechanism for channeling acyl groups into PC, and thereby potentially also regulate oil qualities in those seeds. It was however surprising to find that microsomal preparations from developing Crambe seeds membranes seemed to have barely detectable activity of this enzyme activity, although membranes from rape, another member of the Brassicaceae family, had this activity. Membranes from both species had similar activities in acylation of glycerol 3-phosphate (Fig. 1b). Both Crambe seeds and elm seeds have high amount of fatty acids that are essentially excluded from membrane lipids in their oil, (57 mol % of erucic acid and 79 mol % of caprylic- and capric acids, respectively). Since elm seed membranes had as high GPCAT activity as the membranes from seeds having mainly PC-derived fatty acids in the oil (castor bean and safflower) and Crambe lacked this activity, there seems to be no correlation between the amount of PC-derived acyl groups in the oil and GPCAT activities in the seeds.

The high background activity of GPCAT in the membranes in the absence of added acyl-CoA, even after washing the membranes with buffer containing BSA, is puzzling. It is clear from our experiments that GPC is acylated with added [^14^C]16:0-CoA at both *sn*-positions. Under these incubation conditions we acylate endogenous LPC with non-radioactive 18:1-CoA before we add [^14^C]16:0-CoA and we inhibit acyl exchange between PC and acyl-CoA with added DTNB. This effectively prevented any acyl groups from acyl-CoA to enter PC other than by acylation of added GPC. In castor bean membranes, the GPCAT activity in absence of added acyl-CoA was as much as 25 % of the activity in assays containing acyl-CoA. When we assayed LPC:LPC transacylation (LPCT activities) in (unwashed) safflower microsomal membranes preparations, we could conclude that there can only be very minor acylation of added *sn*-1-[^14^C]acyl-LPC by endogenous acyl-CoA, despite very high LPCAT activity in these membranes and nine times more membranes than in the GPCAT assays. It is therefore obvious that GPC from particularly safflower and castor bean also is acylated with groups from endogenous lipids to a significant extent. At this point we can only speculate which substrates and which enzyme reactions could be responsible for this reaction. It could be hypothesised that this enzyme is the same as the one using acyl-CoA. It has been shown that another plant acyl transferase, the phytol ester synthases in Arabidopsis, could utilize acyl groups from both complex lipids as well as acyl-ACP and acyl-CoA (Lippold et al. 2012). One could also speculate that the LPCT activity can give raise to LPC by its reverse reaction, i.e., PC + GPC → 2LPC. Since neither the reverse of forward reaction of the LPCT uses any net energy, there is no thermodynamic reason to believe that it should not operate in both directions. Another hypothesis would be a choline exchange between GPC and endogenous PC. It is anyhow clear from our experiments that in presence of added acyl-CoA, the main reaction is acylation of added GPC with acyl-CoA. When radioactive 16:0-CoA was used under condition that prevented any acylation into endogenous PC in the absence of added GPC, radioactive PC was efficiently formed upon addition of non-radioactive GPC, excluding both reversibility of LPCT activity and choline exchange in formation of this radioactive PC.

Addition of acyl-CoA to GPCAT assays with [^14^C]GPC and membranes from oil seeds having GPCAT activity resulted in formation of nearly only PC and no LPC except for a small amount in elm microsomes, indicating that the LPCATs could acylate all LPC formed in these membranes. In assays without addition of acyl-CoA, PC was, surprisingly, still the dominating labelled lipid but 25–35 % of the label was also found in LPC. It could be speculated that this PC was formed by forward LPCT activity using the LPC formed by GPCAT, by choline exchange between GPC and endogenous PC and/or by the reverse reaction of the PDAT (Ghosal et al. 2007).

The demonstration of GPCAT activity in yeast, as previously reported (Stålberg et al. 2008), as well as in membranes from Arabidopsis roots and shoots suggests that this enzyme has a role in cellular lipid metabolism that is not necessarily linked to oil producing tissues. A recent study (De Smet et al. 2013) showed that enhanced post-synthetic acyl chain remodelling of PC occurs in yeast overexpressing the glycerol 3-phosphate acyltransferase (*Sct1p*). The exchange of acyl chains occurred at both the *sn*-1 and *sn*-2 positions of PC and required *PLB1* for optimal efficiency and the authors speculated on the involvement of GPCAT in this process.

The substrate for the GPCAT reaction is GPC and the last step in GPC formation is deacylation of LPC. Our results show that when physiological levels of LPC substrates were incubated with safflower microsomes, the main metabolite was PC and that this PC was mainly formed by transacylation of an acyl group from one LPC molecule to another LPC molecule with the concomitant release of GPC. We have termed this activity LPCT (LPC transacylase) activity according to earlier suggestions (Sugimoto and Yamashita 1999). The second most abundant labelled metabolite in the assays was DAG and with all likelihood this lipid was mainly formed from PC by the action of PDCT (Lu et al. 2009; Stobart and Stymne 1985). DAG and PC made up 75 % of the metabolites that used [^14^C]18:1-LPC as a substrate, whereas the remaining 25 % was free fatty acids. Thus, these in vitro data suggest that LPCT activity is at least as important route to GPC production as acyl lipase in safflower membranes. The PC produced through transacylation catalysed by LPCT activity would mainly have acyl groups originating from position *sn*-1 of LPC at its *sn*-2 position of PC. Since about 10 % of the added LPC substrate was *sn*-2-LPC, minor acyl transfer might also occur from this position to both positions *sn*-1 and *sn*-2. We showed that saturated fatty acids are efficiently transferred to the *sn*-2 position of PC by LPCT, although somewhat less efficiently than unsaturated C_18_ fatty acids. Since the levels of saturated fatty acid are very low in the *sn*-2 position of PC in plant cells, it would suggest that LPCT mediated re-synthesis of PC is not a significant contributor to the pool of PC in the cell. However, since acyl groups at the *sn*-2 position of PC are turned over much more rapidly than at *sn*-1 and LPCAT, the major enzyme for re-acylation of the *sn*-2 position with acyl-CoA, discriminates against saturated fatty acids in plants (Lager et al. 2013), these saturated fatty acids at position *sn*-2 might be rapidly replaced.

The amounts of PC produced by LPCT activities were similar with saturated and unsaturated LPC but only 10–25 % of the radioactive DAG formed with the C_18_ unsaturated [^14^C]LPC substrates were formed with saturated substrates in the safflower membranes. Since the formation of radioactive DAG is likely to be catalysed by the PDCT enzyme, it implies that this enzyme has a strong specificity for unsaturated PC species. Previously, Bates et al. (2009) have shown that there are several DAG pools in soy bean seeds, of which only certain pool(s) equilibrate with PC. Our data indicate that there is also separate PC pool(s) or molecular species are involved in this equilibration, at least in the safflower microsomal membranes.

In conclusion, we show that plant membranes have LPCT and GPCAT activities that, working in concert, are able to re-synthesize PC from GPC. The presence of these activities in microsomal membrane from developing oil seeds suggests that they are involved in acyl editing of PC. Acyl groups in PC are rapidly turned over in plant cells, in particular oils seeds having high amount of polyunsaturated fatty acids in the triacylglycerols (Bates et al. 2014). Polyunsaturated fatty acids are synthesised on PC and efficient mechanisms are needed for channelling oleic acid into PC and transfer polyunsaturated fatty acids into TAG. However, not only in oil seeds but also in leaf cells a major part of acyl groups are shunted through PC (Bates et al. 2007). Earlier identified enzymes involved in this acyl editing of PC are LPCATs, PDCTs and PDATs and, possibly, also different phospholipases (Bates et al. 2014). A possible role for GPCAT enzyme activity would be formation of LPC with acyl-CoA produced in the plastid envelope from acyl groups produced de-novo in the plastid. However, Bessoule and co-workers (Bessoule et al. 1995) failed to detect such activity in envelope fraction. The identification of the genes for enzymes capable to carry out GPCAT and LPCT reactions and subsequent studies of deletion mutants and overexpressors are likely to give information regarding the role of these enzymes in overall PC acyl editing in plants. The successful cloning of the PDAT enzyme, first in yeast and subsequently in plants (Dahlqvist et al. 2000; Ståhl et al. 2004), fosters some optimism that this might be feasible, at least for the GPCAT activity.

### **Author contribution**

IL and SS conceived the study. IL, SS, BG and LE conducted the experiments. All authors participated in data analyses and editing of the manuscript. IL and SS wrote the article.

## Abbreviations

PC: Phosphatidylcholine
GPCAT: Acyl-CoA:glycerophosphocholine acyltransferase
LPC: Lysophosphatidylcholine
GPC: Glycerophosphocholine
LPCT: Lysophosphatidylcholine transacylase
CPT: CDP-choline:diacylglycerol cholinephosphotransferase
PLA, PLB: Phospholipase A, B
TAG: Triacylglycerol
LPCAT: Acyl-CoA:lysophosphatidylcholine acyltransferase
PDAT: Phospholipid:diacylglycerol acyltransferase
16:0: Palmitic acid
18:0: Stearic acid
18:1: Oleic acid
18:2: Linoleic acid
18:3: Α-linolenic acid
PDCT: Phospholipid:diacylglycerol cholinephosphotransferase
FFA: Free fatty acid
DAG: Diacylglycerol
TLC: Thin layer chromatography
BSA: Bovine serum albumin
DTNB: Dithionitrobenzoic acid
